#  Conditional accuracy in response interference tasks: Evidence from the Eriksen flanker task and the spatial conflict task

**DOI:** 10.2478/v10053-008-0005-4

**Published:** 2008-07-15

**Authors:** John F. Stins, J. C. Tinca Polderman, Dorret I. Boomsma, Eco J. C. de Geus

**Affiliations:** 1 Department of Biological Psychology, Vrije Universiteit, Amsterdam, the Netherlands; 2 Research Institute MOVE, Faculty of Human Movement Sciences,VU University, Amsterdam, the Netherlands

**Keywords:** response interference, sequential analysis, accuracy, Simon task, flanker

## Abstract

Two well-known response interference tasks are the Eriksen flanker task and the spatial conflict task. The tasks are logically equivalent, and comparable effects of current and previous stimulus type (congruent or incongruent) have been shown with regard to reaction time (RT). Here, we investigated whether interference and sequential trial effects also had comparable effects on accuracy. We specifically tested whether these effects interacted with the speed of responding using conditional accuracy functions (CAFs). The CAFs revealed that in both tasks congruency and sequential trial effects on accuracy are found only in trials with fast responses (< 600 ms). Sequential trial effects on accuracy were weaker for the flanker task than for the spatial conflict task. In very fast trials (< 400 ms) response activation by distracting flankers led to below-chance performance in the flanker task, but response activation by incongruent spatial location did not lead to below-chance performance in the spatial conflict task. The pattern of results hints at subtle differences in processing architecture between the tasks.

## INTRODUCTION

Response interference refers to the finding that performance deteriorates when a dominant response has to be suppressed in order to give the alternate (instructed) response, relative to the condition in which the dominant response and the activated response are the same. An often-studied paradigm is the Eriksen flanker task (e.g., Eriksen & Schultz, 1979), where subjects have to respond to a central target flanked by distractors, usually arrows or letters. When the target arrow and the flanking arrows all point in the same direction (when they are congruent), reaction time is shorter and performance is more accurate than when the target arrow points in a different direction than the flanking arrows, that is, they are incongruent.

The current study is motivated by a study of Gratton, Coles, and Donchin (1992) , who investigated RT and accuracy in the Eriksen flanker task. They suggested that stimulus processing takes place in two phases: first, a brief “quick and dirty” parallel phase, during which all stimulus elements (including the flankers) are processed in parallel, followed by a second, more elaborate, focused phase, in which subjects select a particular location in the visual field for further processing. During the focused phase subjects inhibit (to some extent) the influence of the flankers on response selection. Support for this two-phase model comes from so-called conditional accuracy functions (CAFs), in which the accuracy for a given trial type is plotted as a function of RT. For very short RTs (< 250 ms) performance on incongruent trials was below chance, but as RTs increased, accuracy for this type of trial quickly rose to near perfect levels. Performance on congruent trials, in contrast, was at near-perfect levels of accuracy for each RT-value. This pattern of results suggests a strategy whereby subjects sometimes respond on the basis of evidence accumulated in the parallel phase (i.e., the identity of the flanker elements) and sometimes on the basis of evidence accumulated in the focused phase (i.e., the identity of the target). If subjects respond mainly on the basis of the identity of the flankers, their accuracy will be near-perfect on congruent trials, but well below chance on incongruent trials, because on these trials the flankers signal the alternate response.^1^ 

Moreover, Gratton et al. (1992) found that this pattern of results was modulated by the previous trial type, in that the dip below chance level for incongruent trials only reached statistical significance when the previous trial was congruent, as opposed to another incongruent one. Specifically, there appears to be an advantage (as evidenced by more accurate performance) for congruent trials preceded by congruent trials (cC), and incongruent trials preceded by incongruent trials (iI) relative to cI and iC transitions. In other words, congruency repetition yields somewhat more accurate performance than congruency change. This pattern of results was essentially mirrored in the RT data. Apparently, subjects are more likely to resort to a parallel processing strategy if they had just encountered a congruent trial. This was explained by Gratton et al. (1992) by assuming that subjects changed the emphasis given to the evidence gained during each of the two phases. This change in emphasis is unintentional and varies on a trial-to-trial basis, so that after a congruent trial subjects are more likely to respond on the basis of evidence gained during the parallel phase. If, in contrast, they had just encountered an incongruent trial, subjects are more likely to respond somewhat more cautiously, and base their response on evidence gained during the focused phase.

These so-called sequential dependency effects have been observed in several studies, both in terms of speed and in terms of accuracy of performance (e.g., Nieuwenhuis, Stins, Posthuma, Polderman, Boomsma, & de Geus, 2006). Similar patterns of results have been obtained using a closely related task: the spatial conflict task. The spatial conflict task (sometimes called the Simon task, e.g., Simon & Rudell, 1967; for a review see Simon, 1990) is based on the finding that when a stimulus and a response are spatially congruent (e.g., left hand response to a stimulus presented in the left visual field or to the left ear), RTs are shorter and responses are more accurate relative to when they are incongruent (e.g., one is left and the other is right). For example, if a left response has to be given to a high-pitched tone, and a right response to a low-pitched tone, RT is shorter and performance is more accurate when the emitted response is on the same side as the stimulated ear. In this design, location of the stimulus (in this example, the ear stimulated) is a task-irrelevant stimulus property, that is, uncorrelated with the identity of the stimulus (in this example, the pitch). This interference effect (or Simon effect) is usually attributed to an automatic activation of the response on the same side as the stimulated side. Sequential dependency effects on RT and on error rate have also been demonstrated in the spatial conflict task (Valle-Inclán, Hackley, & de Labra, 2002; Valle-Inclán, Hackley, & MacClay, 1998).

The spatial conflict task and the Eriksen flanker task are logically equivalent, in that in both tasks (a) subjects have to respond to one task-relevant stimulus attribute and try to suppress responding to the other task-irrelevant attribute, (b) the task-irrelevant attribute is sometimes congruent and sometimes incongruent with the instructed response, (c) the task-relevant and task-irrelevant attributes are uncorrelated, and (d) trial congruency varies on a trial-to-trial basis.

However, despite these similarities important differences remain, especially as regards differences in information processing architecture. First, one difference concerns the nature of the attentional movements in both tasks. In the flanker task, upon stimulus presentation attention has to “zoom in” from a higher-order to a lower-order level of representation, that is, attention has to focus on the target (for a more thorough treatment of this issue, see Eriksen & St. James, 1986; Stoffer, 1991). In the Simon task, in contrast, attention has to make a lateral (same-level) shift to the imperative stimulus. These different types of attentional movements may have implications for the temporal dynamics of stimulus code formation (Stoffer, 1991). Second, in the spatial conflict task, the task-relevant and task-irrelevant features belong to different perceptual dimensions, for example color and location. These stimulus features are processed along separate channels (this is known as *dual-route processing*). It is widely assumed that stimulus identity is processed along a controlled route, whereas location is processed automatically along an unconditional route. In the Eriksen flanker task, in contrast, the flow of information proceeds along the same channel (see also Wendt, Kluwe, & Peters, 2006). Related to this, the information of the flankers outweighs the information of the target because the flanker information constitutes more visual elements (usually four) than the target (one). As such, information processed in the parallel phase tends to be dominated by the flankers (Gratton et al., 1992). Finally, in the flanker task the target arrow on a given trial may become a distractor arrow on the next trial, which may give rise to a mechanism of negative priming (e.g., Nieuwenhuis et al., 2006; Ullsperger, Bylsma, & Botvinick, 2005), whereas negative priming is unlikely in the spatial conflict task.

The differences between the tasks, in turn, have led to different accounts of sequential dependency effects in the respective tasks. In a nutshell, sequential dependencies in the Eriksen flanker task have traditionally been explained by the conflict-control loop theory, according to which the response conflict induced by the incongruency between target and flankers on trial n leads to a temporary increase in cognitive control on trial n+1, resulting effectively in a reduction of flanker interference following incongruent trials (e.g., Botvinick, Nystrom, Fissell, Carter, & Cohen, 1999). However, more recently it was argued that sequential effects in the flanker task can in effect be explained by the subset of trials that exhibit exact stimulus-response repetitions (Mayr, Ahw, & Laurey, 2003; Nieuwenhuis et al., 2006), so that a mechanism of associative priming may account for the sequential dependency effect. With respect to the spatial conflict task it is generally assumed that sequential dependency effects arise within the context of a dual-route model of information processing. According to this model, identity of the target (e.g., its shape or color) is processed along an intentional control route that in effect realizes the task instruction. The task-irrelevant stimulus attribute (i.e., its location) is processed in an automatic fashion along the unconditional route and directly activates the ipsilateral response. Within this model, sequential dependency effects are explained by selective gating and/or suppression of these routes as a function of the congruency level of the preceding stimulus, leading to trial-by-trial changes in activation that bias processing of the current trial (e.g., Stürmer, Leuthold, Soetens, Schröter, & Sommer, 2002). However, this account was recently challenged by Hommel and coworkers (e.g., Hommel, Proctor, & Lu, 2004), who favor a so-called *feature integration account*. According to this account there is a processing advantage for trial sequences involving exact stimulus-response repetitions (same identity and same location) and trial sequences where both the stimulus and the response alternate (different identity and different position) relative to where just one of the stimulus attributes changes and the other remains the same. This processing advantage in effect leads to an advantage of cC trials over iC trials, and iI trials over cI trials, both in terms of speed and in terms of accuracy.

So there is considerable interest in sequential dependency effects in both tasks, and in the extent to which they share similarities in their information processing architectures. But to our knowledge no direct tests have been performed comparing sequential effects in both tasks within the same subject group. The aim of this study is to directly compare accuracy scores obtained with both tasks, regarding (a) repetition effects and (b) the dynamics of direct activation. With respect to repetition effects, sequential dependency effects on RT have been repeatedly demonstrated in both tasks (see above), although the effect seems to be somewhat stronger in the spatial conflict task than in the flanker task. In this study we test whether a comparable pattern of sequential trial effects can be found for the accuracy data in both tasks, and whether sequential dependency effects on accuracy are indeed stronger in the spatial conflict task than the flanker task. Our second interest – temporal dynamics of direct activation – will be investigated using the CAFs as described above. By constructing CAFs for both tasks, it becomes possible to examine whether the time course of activation of task relevant and task irrelevant stimulus attributes in the spatial conflict task is comparable to the time courses of activation in the flanker task. More specifically, we will test whether in the spatial conflict task subjects will base their fast responses not on the identity of the stimulus but on its left or right location. If this is so, then we expect to find below-chance accuracy for the very fast incongruent spatial conflict trials, just as was found for the flanker task.

To this end we reanalyzed a set of flanker data published in Nieuwenhuis et al. (2006) . In their Experiments 1 to 5 an attempt was made to disentangle associative stimulus-response priming from conflict-driven adaptations in cognitive control in the flanker task. The subject group in their Experiment 5 (but not the other experiments) consisted of a large number of monozygotic twins, dizygotic twins, and their siblings, with a mean age of 12 years. The children were recruited from the Netherlands Twin Register (Boomsma, 1998).^2^

The current study differs in three regards from Experiment 5 in the Nieuwenhuis et al. (2006) study. First, that study asked a theoretical question that was quite different from ours, namely whether sequential trial effects are due to (low level) priming, or due to (top-down driven) adaptations in cognitive control. However, in the present study, the emphasis is on the temporal dynamics of activation. Second, the Nieuwenhuis et al. (2006) study looked only at performance on the flanker task, whereas in the current study we also analyzed Simon data (not reported in Nieuwenhuis et al., 2006), which permits a direct comparison between the processing architecture of the tasks for the reasons outlined above. Third, in Experiment 5 of Nieuwenhuis et al. (2006) the experimental group consisted of twins. However, it could be the case that pairs of twins (especially monozygotic ones) are alike in their performance, so that strictly speaking the observations are not independent. To that end, we decided to randomly select one twin from each pair and analyze only those data. 

## METHOD

### Participants

The subject group consisted of 137 12-year-old children. Although this age group is younger than the subject groups often used in experimental psychology (usually first-year undergraduates), there is evidence that at this age cognitive functions such as attentional control have already reached maturity (e.g., Ridderinkhof, van der Molen, Band, & Bashore, 1997). The children were all twins and were randomly selected from a group of 137 pairs of twins, so as to exclude possible high phenotypic intercorrelations. Pairs of twins were first asked in writing whether they were willing to participate in the study. Permission was also asked of the parents or guardians. If permission was granted, the families received further information on the study and were invited to come to the campus site to do the tests. On the day of testing, both the children and their parents or legal representatives signed an informed consent form.

### Procedure

The children performed a range of neuropsychological tests that were administered in the same order. Short breaks were given between tests. The entire session lasted approximately 4 hrs per child. The spatial conflict task and the Eriksen flanker task were performed on a computer. Subjects were seated in front of a computer monitor and a panel of two response buttons (left and right). The monitor and the response buttons were approximately aligned with the vertical meridian of each participant’s body.

In the spatial conflict task subjects were first presented with a white fixation cross for 500 ms. Immediately after the cross had disappeared a red or a green disk (1.9 cm in diameter) appeared for 500 ms, either left or right from fixation. The distance between the fixation cross and the inner edge of the disk was 2.5 cm. Stimulus color and stimulus location were uncorrelated. Subjects were instructed to press the left key in response to a green disk and the right key in response to a red disk, regardless of stimulus location. Subjects received a total of 120 trials (60 red stimuli and 60 green stimuli) in random order. Half of each stimulus type was presented left, and the other half was presented right. The trials on which the stimulus location happens to be on the same side as the required response are the congruent trials; the other trials are the incongruent ones. Prior to the experiment subjects received 12 practice trials that were not analyzed. The spatial conflict task lasted approximately 10 min.

In the Eriksen flanker task subjects were first presented with a white fixation cross for 500 ms, which was immediately followed by a horizontal array of five equally sized and spaced white arrows for 800 ms. The array was 10.5 cm wide. Subjects were instructed to attend to the central arrow and ignore the four flankers. Subjects were to press the left key for a left facing central arrow and the right key for a right facing central arrow. The flanking arrows either all pointed in the same direction as the target arrow (e.g., “< < < < <”), or they all pointed in the opposite direction (e.g., “< < > < <”). The trials on which the flanking arrows pointed in the same direction as the target arrow were the congruent trials; the trials in which they pointed in the opposite direction were the incongruent trials. Subjects received a total of 80 trials (40 congruent and 40 incongruent ones) in a random order, requiring an equal number of left or right responses. Prior to the experiment subjects received 12 practice trials that were not analyzed. The Eriksen flanker task lasted approximately 10 min.

### Data analysis

For both tasks, each trial was classified according to its congruency (C or I) and the congruency of the previous trial (c or i), yielding four unique transitions. We performed a 2 × 2 × 2 ANOVA on the percentages correct with trial type (congruent vs. incongruent), previous trial type (congruent vs. incongruent), and task (flanker or Simon) as factors. We applied the following restriction: If there were two or more consecutive errors, we only included the first one and we did not analyze the consecutive one(s). We also examined repetition effects in the RT data to rule out possible speed-accuracy trade-offs.

For construction of the CAFs, we first classified each subject’s RTs into 100-ms bins. For each subject and for each transition we computed the mean percentage of correct responses in each bin. These percentages were then averaged across all subjects, resulting in the CAFs. Although in principle one could construct CAFs spanning the entire RT-range, an upper and lower limit to the RT-bins was set. The upper limit was motivated by our observation that RTs greater than 600 ms for all conditions had reached near-perfect levels of accuracy and were no longer informative. We will therefore plot, for both tasks, CAFs only up to the > 700 ms-bin. The lower limit was deemed necessary because initial inspection of the results revealed that extremely fast RTs were very rare. So if, for example, there was only one subject who made a response below 100 ms, and if this happened to be the correct response, the CAF would give an accuracy level of 100% for this bin, which is clearly nonsensical. We decided to construct CAFs for the flanker task starting from the RT-bin of 300 to 400 ms, and for the spatial conflict task for the RT-bin of 200 to 300 ms. Observations below these boundaries were considered too infrequent; for the flanker task there were 27 errors out of a total of 2610 (1.03%) in the 100 to 300-ms RT range, whereas for the Simon task there were 21 errors out of a total of 2800 (0.75%) in the 100 to 200-ms range.

## Results

**Figure 1.**
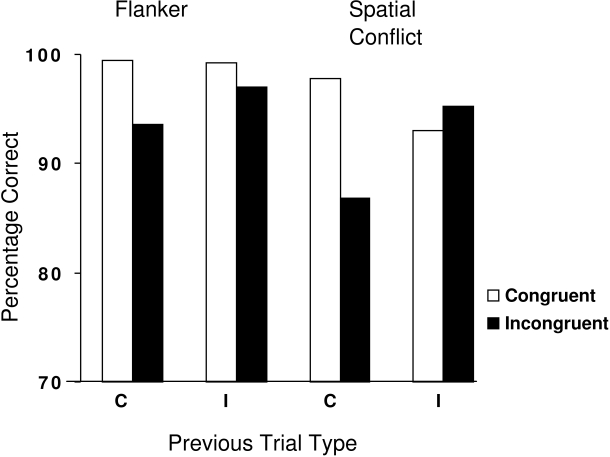
Mean percentage of correct responses for the flanker task and the spatial conflict task for congruent and incongruent trials, as a function of the preceding trial type (C = Congruent, I = Incongruent).

Due to technical problems the Simon data of 5 subjects and the flanker data of 3 different subjects were not stored on the computer. In addition, the flanker data of 1 subject were discarded due to an extremely high error rate (63% errors in the incongruent condition). The mean percentages correct for both tasks, as a function of trial type and previous trial type are shown in Figure 1.

The ANOVA revealed the following effects: There was a main effect of task, *F*(1, 128) = 121.3, *p* < .001, indicating that performance on the flanker task was more accurate than performance on the Simon task (97 vs. 93% correct, respectively). Next, there was a main effect of trial type, *F*(1, 128) = 118.2, *p* < .001. This was due to the expected effect of trial type: Accuracy was higher with congruent trials than incongruent ones (97.3 vs. 93.1%). Also the main effect of previous trial type was significant, *F*(1, 128) = 31.4, *p* < .001. This effect indicates that accuracy for a given trial was higher when the previous trial was an incongruent one than a congruent one. Two interactions were significant: The two-way interaction of trial type and previous trial type was significant, *F*(1, 128) = 125.8, *p* < .001, which was modulated by the three-way interaction of task, trial type, and previous trial type, *F*(1, 128) = 55.9, *p* < .001. The two-way interaction indicates that cC trials were more accurate than iC trials, and that iI trials were more accurate than cI trials. In other words, congruency repetition leads to more accurate performance than congruency change.

The three-way interaction suggests that this benefit was task dependent, which we tested by performing separate ANOVAs for each task. For the flanker task the two-way interaction of trial type and previous trial type was significant, *F*(1, 132) = 25.7, *p* < .001. Using a post-hoc test we found that iI transitions were more accurate than cI transitions, *T*(132) = 5.45, *p* < .001, but that cC transitions were not more accurate than iC transitions ( *p* > .1). For the spatial conflict task the same interaction was also significant, *F*(1, 131) = 131.8, *p* < .001. Using a post-hoc test we found that iI transitions were more accurate than cI transitions, *T*(131) = 9.54, *p* < .001, and also that cC transitions were more accurate than iC transitions, *T*(131) = 8.35, *p* < .001. Another way of looking at this interaction is by examining what happens to the benefit of congruent trials over incongruent ones when in both cases the previous trial was incongruent. Using a paired t-test, we found in the flanker task the expected higher accuracy for congruent trials than for incongruent trials when they were preceded by an incongruent trial, *T*(132) = 5.58, *p* < .001. However, this effect was reversed for the spatial conflict task: Congruent trials resulted in lower accuracy than incongruent trials when preceded by an incongruent trial, *T*(131) = 3.52, *p* < .001. In sum, congruency repetition yielded overall more accurate performance, and this effect was more prominent in the spatial conflict task than the flanker task.

**Figure 2.**
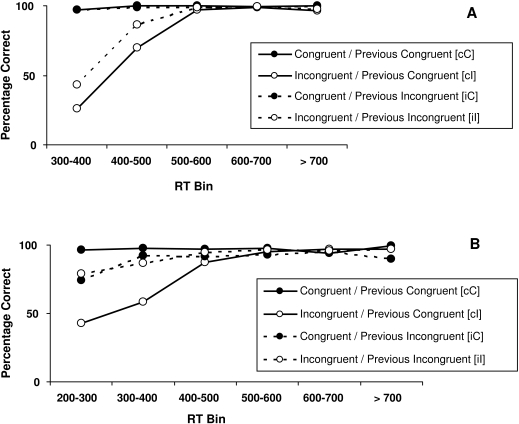
Mean percentage of correct responses as a function of RT-bin, for the flanker task (A, top) and the spatial conflict task (B, bottom), for each of the four congruency transitions.

The temporal dynamics of these effects can also be seen from the conditional accuracy functions, shown in Figure 2a (flanker task) and Figure 2b (spatial conflict task). First, as can be seen from both figures, accuracy sharply increased with increasing RT, attaining near-perfect levels at about 600 ms. Thus, the observed effects of trial type and previous trial type on accuracy originate mainly in the fast RT-regions. Second, the tasks differed with respect to congruency repetition effects. For the spatial conflict task, congruency repetition yielded unambiguously more accurate performance on fast trials than congruency change. For the flanker task, however, only iI transitions yielded better performance than cI transitions, whereas there was no difference between iC and cC transitions. Third, for the flanker task the accuracy for incongruent trials that are preceded by congruent trials obtained with the fastest RT bin is 26.2%, which is well below chance level, *T*(41) = 3.81, p < .001. The accuracy level for iI trials at the same RT bin is 43.4%, which did not statistically differ from 50%. For the spatial conflict task, in contrast, accuracy for the cI trials at the fastest bin does not drop below chance level. The accuracy level for this subcondition is 46%, which is not statistically different from 50%.

The ANOVA on the RTs revealed a two-way interaction of trial type and previous trial type, indicating an overall congruency repetition benefit, *F*(1, 128) = 144.9, p < .001. This interaction was modulated by the three-way interaction of task, trial type, and previous trial type, *F*(1, 128) = 23.0, *p* < .001. Examination of the cell means revealed this was due to the fact that the congruency repetition benefit was larger for the spatial conflict task than for the flanker task (38 vs. 19 ms, see Table 1). Another way of looking at the data is in terms of the size of the reduction of the congruency effect, following an incongruent trial. For the flanker task the congruency effect dropped from 122 ms (following a congruent trial) to 84 ms (following an incongruent trial), resulting in a net reduction of 38 ms. For the Simon task, however, the reduction of the congruency effect was nearly twice as large, dropping from 69 ms to 6 ms, resulting in a net reduction of 75 ms. The overall pattern of RTs is quite similar to the accuracy scores, especially as regards repetition scores. This indicates that there is no reason to suspect subjects had engaged in a speed-accuracy trade-off.

**Table 1.** Reaction time (in miliseconds; standard errors in parentheses) as a function of task (flanker vs. spatial conflict), trial type, and previous trial type.

Task		Flanker		Spatial conflict	
Previous trial type		Congruent	Incongruent	Congruent	Incongruent
Trial type	Congruent	552 (8)	574 (10)	452 (6)	498 (8)
	Incongruent	674 (10)	658 (10)	521 (6)	492 (7)

## Discussion

In this experiment a group of 137 children, aged 12, performed two well-known response interference tasks: the spatial conflict task and the Eriksen flanker task. Both tasks involve an easy (congruent) condition and a more difficult (incongruent) condition. Congruent conditions yielded more accurate performance than incongruent conditions, but only in trials with fast response speed (RTs < 600 ms). In addition, in both tasks we found that the probability of producing a correct response for a given trial was somewhat higher when subjects had just encountered an incongruent trial than a congruent one, again only with the fast responses. Furthermore, congruency repetition (cC and iI trials) resulted in overall more accurate performance than trials involving congruency change (cI and iC). The congruency repetition effect on accuracy was also more pronounced in the spatial conflict task than in the flanker task. This benefit was due to the fact that in the spatial conflict task both cC transitions and iI transitions were more accurate than their counterparts (cI and iC), whereas in the flanker task only cC transitions resulted in superior performance.

These accuracy data mirror previous findings on sequential trial effects on RT that were also larger in the spatial conflict task (Gratton et al., 1992; Valle-Inclán, Hackley, & McClay, 1998; Valle-Inclán et al., 2002). Apparently, subjects’ level of processing selectivity is not constant across the experiment, but fluctuates on the basis of the preceding trial. More specifically, after an incongruent trial subjects tend to pay more attention to the task-relevant stimulus, resulting in fewer errors during fast responses, whereas after a congruent trial subjects are more prone to base their response on task-irrelevant information (location or flankers), yielding somewhat more errors. Furthermore, subjects benefit more from such modifications in processing selectivity in the spatial conflict task than in the flanker task.

With respect to the profiles of the CAFs, the tasks yielded somewhat diverging results. For the flanker task, we observed the predicted accuracy drop below 50% for the very fast incongruent trials that were preceded by congruent trials, which indicates that subjects base their fast responses on the identity of the flankers, instead of the target. This results in below chance performance on incongruent trials, whereas performance on the very fast congruent trials is already near-perfect. For the spatial conflict trials, however, accuracy never dropped below chance level. Even at the fastest RTs subjects do not base their response on the task-irrelevant location of the stimulus.

This clear cut task difference contrasted with the expectations derived from the two-phase stimulus processing model for interference tasks proposed by Gratton et al. (1992) . This model would have predicted below-chance performance on fast responses in both tasks, at least when the interference effect in both tasks occurs in the same processing steps. The discrepancy between the tasks could be due to differences in the way task-irrelevant information is processed. In the flanker task the flankers are always processed in parallel with the target along the same channel, as put forward by the continuous flow model of information processing (e.g., Gratton et al., 1992). Initially, flanker information is more dominant and directly primes the corresponding response output. If subjects have just encountered a congruent trial they are likely to emit their response based on this parallel phase, leading to fast errors. With the spatial conflict task, in contrast, target information (identity) and task irrelevant target location are processed along separate routes that converge at the response selection stage. Based on the congruency level of the preceding trial, the automatic route will either receive extra activation (when the previous trial was congruent), or the route will be temporarily suppressed (as when the previous trial was incongruent). In other words, response activation and repetition effects seem to occur more downstream in the flanker task than in the spatial conflict task. This hypothesis is supported by electromyographic (EMG) studies that examined the stage at which conflict arises. Burle, Possamaï, Vidal, Bonnet, and Hasbroucq (2002) argued that in the flanker task response conflict may occur at the level of the peripheral motor system, whereas in the spatial conflict task conflict seems to be localized more upstream. 

In sum, we have demonstrated that accuracy data on the spatial conflict and flanker task share important similarities. This is in line with recent data from brain imaging studies (Fan, Flombaum, McCandliss, Thomas, & Posner, 2003) that found that these tasks engage the same region of the anterior cingulate cortex. Furthermore, Kunde and Wühr (2006) investigated sequential modulations in the spatial conflict task and the Eriksen flanker task, and based on their analyses they concluded that the tasks share important control functions. We replicated the evidence for below-chance performance in the early processing stage in the flanker task, where the incongruent flankers seem to dominate the motor response tendency, at least when preceded by a congruent trial. In the spatial conflict task, no such below-chance performance in the early processing stage was found. Despite their similarities, subtle differences remain in these tasks in the nature of interference and sequential trial effects. We argue that these differences are likely due to whether task-irrelevant information is processed along the same route as the target (as in the flanker task) or along a separate route, as in the spatial conflict task.

## Footnotes

1 Actually, Gratton et al. (1992) identified an additional third phase, even prior to the parallel phase: As soon as the visual onset reaches threshold, subjects can in principle emit a response in the complete absence of information on the identity of the elements. Subjects thus simply guess, meaning that – on the extremely fast RTs – accuracy on both congruent trials and incongruent ones is at chance level. But given that this type of response is extremely rare, we will not deal with this “guessing phase” in the present study.

2 The purpose of examining twin data is that it allows one to test whether differences in a given trait (cognitive or behavioral) are due to genetic differences or environmental differences, such as upbringing. By examining the patterns of twin correlations on a range of measures, it is possible to quantify the heritability of a given trait, which can be defined as the proportion of phenotypic variance that is explained by genetic variance (for details, see Plomin, DeFries, McClearn, & McGuffin, 2001). In the current study and in the Nieuwenhuis et al. (2006) study, we did not examine individual differences in cognitive performance for the following reason: Many measures that are of interest to cognitive psychology are based on difference scores, for instance, the time difference or accuracy difference between two or more conditions. However, a psychometric property of difference scores (such as the size of the flanker effect) is that they tend to have low reliabilities. This, in turn, results in low twin correlation values, which thus limits the use of difference scores in genetic analysis (see also Stins, Polderman, Boomsma, & de Geus, 2005).
